# Dual targeting micelles loaded with paclitaxel and lapatinib for combinational therapy of brain metastases from breast cancer

**DOI:** 10.1038/s41598-022-06677-8

**Published:** 2022-02-16

**Authors:** Heng Lu, Tianran Chen, Yiran Wang, Yuwei He, Zhiqing Pang, Yajie Wang

**Affiliations:** 1 Department of Oncology, Shanghai Pudong New Area People’s Hospital, Shanghai, 201200 China; 2 Department of Oncology, Changhai Hospital Affiliated To Navy Medical University, Shanghai, 200433 China; 3 Department of Pharmaceutics, School of Pharmacy, Fudan University and Key Laboratory of Smart Drug Delivery, Ministry of Education, Shanghai, 201203 China

**Keywords:** Breast cancer, Drug discovery, Nanoscience and technology

## Abstract

Due to the presence of the blood–brain barrier (BBB), the delivery of general drugs into the brain tissue remains to be a tricky problem. For patients with brain metastases from breast cancer, drug delivery systems must overcome this physical barrier. Targeted nano vehicles arise as a promising alternative to deliver drugs to brain tissues successively. Herein, a dual targeting micelle drug delivery system loaded with paclitaxel (PTX) and lapatinib (LPTN) was developed for combinational therapy of brain metastases. In our study, it was shown the micelles modified with Angiopep-2 had high loading efficiency of paclitaxel and lapatinib (Ang-MIC-PTX/LP). In addition, Ang-MIC-PTX/LP could transport across the in vitro BBB model and accumulate in breast cancer cells. After intravenous injection, Ang-MIC significantly accumulated in the brain metastasis. Ang-MIC-PTX/LP could also extend the life span of brain metastasis mouse models. Overall, this study provided a promising method for treatment of brain metastases from breast cancer.

## Introduction

Breast cancer is the most common malignancy in women and it was reported one in eight women are diagnosed with invasive breast cancer in her lifetime^1^. According to statistics in 2012, the death rate of breast cancer accounted for the fifth of all tumors, which was 25% of all new cancer cases in the world^2^. As a biomarker in human malignancies, human epidermal growth factor receptor-2 (HER-2) is overexpressed in 25–30% of breast cancer cases. The overexpression of HER-2 is closely related to a high degree of aggressiveness, poor prognosis, more incidence to relapse, and a greater risk of brain metastases, leading to worse prognosis and shorter survival time^3^.

Paclitaxel (PTX) is an effective broad-spectrum anti-neoplastic agent. Particularly, it plays a significant role in the treatment of breast cancer, ovarian cancer, head and neck cancer, and non-small cell lung cancer^4^. However, HER-2-positive breast tumor signifies easy resistance to chemotherapy drugs including PTX^5^. HER-2 targeted drugs, including trastuzumab and lapatinib (LPTN), are able to suppress the dimerization and kinase activity of HER2-positive cancers, resulting in significantly improved clinical outcomes in HER-2-positive breast cancer patients. As a tyrosine kinase inhibitor that targets both epidermal growth factor receptor (EGFR) and HER-2^6^, LPTN could not only inhibit the proliferation of HER-2 over-expressing breast cancer^7,8^, but also significantly enhance the sensitivity of PTX-resistant patients to PTX^9^. Furthermore, combination treatment with LPTN and PTX has shown synergistic effects compared with PTX alone in patients with HER-2-positive breast cancer in phase II and III clinical trials^9–11^.

However, both LPTN and PTX have a poor water-solubility, particularly LPTN, which makes them difficult to prepare formulations suitable for intravenous injection^12^. To address this problem, amphiphilic micelles were developed for drug delivery. The hydrophobic drugs are wrapped in the hydrophobic core of polymer, while the hydrophilic polymer shell provides solubility and stability in water^13^. In addition, the nano-size polymeric micelles allows for passive drug targeting based on the enhanced permeability and retention (EPR) effect at leaky tumor tissues^14^.

Unfortunately, about half patients with HER-2 positive metastatic breast cancer suffered from brain metastasis. The BBB is the most important barrier in brain-targeted delivery. Drug delivery systems (DDS) for brain tumor targeting should overcome multiple physical barriers besides the BBB. The ideal DDS should cross the BBB without harming its integrity, target cancer cells inside the brain, and release the therapeutic agents.

Angiopep-2, a 19-amino-acid peptide, is one of the vectors designed to target the low-density lipoprotein receptor-related protein (LRP) to cross the BBB^15,16^. For instance, Angiopep-2-PTX conjugate, named ANG1005, has been approved by the FDA for the phase III clinical study of brain metastase from breast cancer, in 2018. In this study, a dual targeting micelle DDS loaded with paclitaxel and lapatinib was developed. Micelles made of poly(ethylene glycol)-poly(lactide) (PEG-PLA) was further modified with Angiopep-2 (Ang-MIC) to achieve both brain targeting and tumor targeting simultaneously. The brain delivery and brain metastases targeting ability of the vehicle was investigated both in vitro and in vivo. After loading with paclitaxel and lapatinib, the anti-brain metastases efficacy of Ang-MIC was also assessed both in vitro and in vivo.

## Materials and methods

### Materials

PTX was purchased from Fujian Southern Pharmaceutical Company, Ltd. (Fujian, China). LPTN was obtained from Hangzhou RongDa Pharmaceutical Chemical Company, Ltd. (Hangzhou, China). Methoxy poly(ethylene glycol)-poly(lactide) copolymer (mPEG2000-PLA2000) was purchased from Jinan Dai Gang Biological Engineering Company, Ltd. (Jinan, China). COOH-PEG2000-PLA2000 was obtained from Shanghai Seebio Biological Company, Ltd. (Shanghai, China). Coumarin-6, 4′,6-diamidino-2-phenylindole (DAPI), 1-Ethyl-3-(3′-dimethylaminopropyl) carbodiimide (EDC), and N-Hydroxysuccinimide (NHS) were all obtained from Sigma (Saint Louis, Missouri). Angiopep-2 was supplied by Chinese Peptide (Hangzhou, China). Dulbecco’s modified Eagle medium (DMEM; high glucose) cell culture medium, fetal bovine serum (FBS), penicillin–streptomycin, and 25% (w/v) trypsin–0.03% (w/v) EDTA solution were purchased from Gibco BRL (Gaithersberg, Maryland, USA). All the other solvents were of analytical or chromatographic grade.

### Cells and animals

Brain capillary endothelial cells (BCECs) and 4T1 cells (mouse breast cancer cells) were kindly gifted by Prof. Xinguo Jiang, School of Pharmacy, Fudan University (Shanghai, China). Human breast cancer cells, SKBr-3 (HER-2 positive, were kindly gifted by Prof. Zhimin Shao, Department of Breast Surgery, Shanghai Cancer Hospital (Shanghai, China). SKBr-3 cells, 4T1 cells and BCECs were all cultured at 37 °C with 5% CO_2_ under fully humidified conditions. Cells were cultured in Dulbecco’s modified eagle medium (DMEM), or Roswell Park Memorial Institute 1640 (1640) supplemented with 10% fetal bovine serum (FBS), 100 IU/ml penicillin, and 100 μg/ml streptomycin sulfate.

Balb/c nude mice (20 ± 2 g) were purchased from the Shanghai Slac Lab Animal Ltd. (Shanghai, China) and housed under standard conditions with free access to food and water. All animal experiments were conducted in accordance with protocols evaluated and approved by the Ethics Committee of Fudan University.

### Methods

#### Preparation of PEG–PLA micelles of LPTN and PTX (MIC-PTX/LP)

60 mg of mPEG–PLA, 4 mg of LPTN and 2 mg of PTX were dissolved in 10 ml of methanol. The solution is prepared into a solid film. Dissolve the film thoroughly with distilled water.The obtained clear micellar solution was then filtrated using a 0.22 μm needle filter to remove the unentrapped PTX and LPTN.

#### Preparation of Angiopep-2 modified PEG–PLA micelles of LPTN and PTX (Ang-MIC-PTX/LP)

The micelles were prepared from 60 mg of PEG–PLA, 6 mg of COOH-PEG–PLA, 4 mg of LPTN and 2 mg of PTX. 4 ml micelle solution, 11.8 mg NHS and 3 mg EDC were incubated at room temperature for 0.5 h for activation, and then 1.5 mg Angiopep2 was added. The solution was incubated at room temperature for 6 h. Activator and free angiopep-2 were removed by ultrafiltration.

#### Characterization of MIC-PTX/LP and Ang-MIC-PTX/LP

##### Drug-loading capacity and encapsulation efficiency

MIC-PTX/LP and Ang-MIC-PTX/LP were diluted in methanol to release PTX and LPTN, respectively. After precipitating the polymers with methanol, samples were filtrated through a 0.22 μm nylon filter. The concentration of PTX in the filtrate was determined via the High Performance Liquid Chromatography (HPLC) analysis method with UV detection at 228 nm (Agilent1200, Agilent, USA). Samples were detected using acetonitrile–water (55:45) as the mobile phase at a flow rate of 1.2 ml/min.

For the other one, LPTN, samples were detected using acetonitrile–potassium dihydrogen phosphate solution (pH = 6.2) (65:35) as the mobile phase at the flow rate of 1 ml/min. The detection wavelength was at 265 nm.

Loading capacity (LC) and encapsulation efficiency (EE) were calculated as indicated below.

1 $$ {\text{LC }}\left( \% \right) \, = \, \left( {{\text{weight of the drug in micelles}}/{\text{weight of the feeding polymer and drug}}} \right) \times {1}00\% $$

2 $$ {\text{EE }}\left( \% \right) \, = \, \left( {{\text{Weight of the drug in micelles}}/{\text{weight of the feeding drug}}} \right) \times 100\% $$

##### Particle size, zeta potential, and morphology

The hydrodynamic particle size and zeta potential of the micelles were measured using a Malvern Zetasizer Nano ZS (Malvern, Worcestershire, UK). All of the dynamic light scattering (DLS) measurements were performed at 25 °C in triplicate. The morphology of MIC-PTX/LP and Ang-MIC-PTX/LP were observed by a transmission electron microscopy (TEM) (Jem-2011, JEOL, Japan) after negative staining with phosphotungstic acid solution (2%, w/v).

##### Drug release of Ang-MIC-PTX/LP

The release of PTX and LPTN in vitro were evaluated by dialysis method. Briefly, 1 ml of ANG-MIC-PTX/LP (containing 1 mg of PTX or 1 mg of LP), 1 ml of free PTX or 1 ml of free LP were introduced into a dialysis bag (MWCO = 13,000 Da; Greenbird Inc., Shanghai, China) and submerged fully into 150 ml of phosphate buffered saline (PBS) with 0.5% Tween 80 at 37 °C with stirring at 100 rpm, respectively. Samples were withdrawn and replaced with an equal volume of fresh medium at appropriate time intervals (0.5, 1, 2, 4, 8, 12, 24, 48, 72 and 96 h). PTX and LPTN released were detected using the HPLC method.

##### Stability of Ang-MIC-PTX/LP and MIC-PTX/LP

To evaluate the stability of the formulations, the ANG-MIC-PTX/LP and MIC-PTX/LP were suspended in PBS and stored at 4 °C. The particle size was measured daily for a week (n = 3).

##### Determination of Angiopep-2 in ANG-MIC-PTX/LP

The BCA (bicinchoninic acid) protein quantification method was used to confirm the presence of the targeting molecule on the surface of the micelle. The amount of targeting molecules in the targeted micelles was also determined. Briefly, free Angiopep-2 (Mw 23,000) was removed by centrifugation using an ultrafiltration tube with a molecular weight cutoff of 30,000. According to the protocol in the BCA protein assay kit, standard gradient reagent and appropriate concentration of ANG-MIC-PTX/LP micelle solution was added to the 96-well plate, and three replicate wells were set for each concentration. After incubation with working reagent at 37 °C for 30 min, the absorbance of samples at 570 nm were measured using a plate reader. A standard curve was drawn to obtain a linear relationship between absorbance and protein content. The concentration of Angiopep-2 in ANG-MIC-PTX/LP was calculated.

#### In vitro cell study

##### BBB penetration ability of Ang-MIC

The BBB model was established in vitro as described previously^17^. Brain capillary endothelial cells were seeded on the inner surface of collagen-coated Transwell inserts (6.5 mm diameter, 0.4 μm pore size; Corning, NY), and SKBr-3 cells were seeded in the lower 24-core plates. Fresh culture medium was changed every 24 h. To verify cell monolayer normally confluency and cell barrier integrity, endothelial cell growth was observed by light microscopy and trans-endothelial electrical resistance (TEER) was determined using Millicell ERS-2 (Millipore, USA). The resistance values of the upper and lower levels reaching 321 ± 13Ω × cm^2^ met the BBB model requirements after 3 days of growth as reported before^17^. We added 100 μl (100 ng/ml) of coumarin-6 loaded blank micelles (Ang-MIC-cou-6 and MIC-cou-6) and 100 μl of fresh culture medium to the transwells bottom dishes separately and incubated at 37 °C for 4 h. Culture medium was removed from the lower culture plate, and cells were then carefully washed for three times with phosphate buffer saline (PBS) and fixed with 4% paraformaldehyde for 15 min.

##### Cytotoxicity study

The cell viability was evaluated using an MTT assay. The 4T1 cells (HER-2 negative) and SKBr-3 cells (HER-2 positive) were incubated with different concentration of ANG-MIC-PTX/LP and MIC-PTX/LP at 37 °C for 24 h. Those treated with the culture medium were used as negative control. After treatment, cell viability was quantitated by determining optical absorption at 490 nm and the negative control was defined as 100% viability. The viability percent of samples was calculated as the percentage of absorbance of the study group over the control group.

#### In vivo study

##### Brain targeting ability of Ang-MIC

The breast tumor-bearing nude mice models were developed as previously described^18,19^. Briefly, 2 × 10^5^ Skbr-3 cells in 10 μl of PBS were inoculated in to the right striatum (2 mm of lateral margin and 4 mm of depth) of nude mice by using a stereotactic fixation device with a mouse adaptor. After 30 days, the model mice were administrated with DiR-labeled PPM (DiR-MIC- or DiR-Ang-MIC, 0.5 mg/kg) via the tail vein. The in vivo fluorescence imaging of mice was then acquired using the in vivo IVIS spectrum imaging system (PerkinElmer, USA) at predetermined time points (3 h, 6 h, 12 h and 24 h) (n = 3).

##### Biodistribution of Ang-MIC

After 24 h, the nude mice were sacrificed and major organs were collected. The fluorescence content of each organ was measured as above mentioned.

#### Therapeutic effect of Ang-MIC-PTX/LP

Breast tumor-bearing mice were randomly divided into three groups (n = 8). Mice were intravenously injected every other day with saline (control), MIC-PTX/LP or Ang-MIC-PTX/LP for a week, respectively. The dose of PTX was 2.5 mg/kg and the dose of LPTN was 5 mg/kg. The survival of the mice was monitored and analyzed.

#### Statistical analysis

All data were reported as the mean ± SD (n = 3). Statistical analysis of all data was analyzed by GraphPad 6.02. One-way ANOVA analysis and post-hoc tests were used for comparison between individual groups. Survival was recorded and analyzed using the log-rank test. The p value less than 0.05 was considered statistically significant.

### Statement on ARRIVE guidelines

The study was carried out in compliance with the ARRIVE guidelines.

## Results

### Characterization of MIC-PTX/LP and Ang-MIC-PTX/LP

The average sizes of MIC-PTX/LP and of Ang-MIC-PTX/LP were 16.23 ± 0.75 and 18.80 ± 0.97 nm respectively (p < 0.01) DLC of 2.9 ± 0.8% and 6.82 ± 1.16%, EE of 95.97 ± 3.47% and 91.21 ± 2.83% for PTX and LPT, respectively.

**Figure 1:**
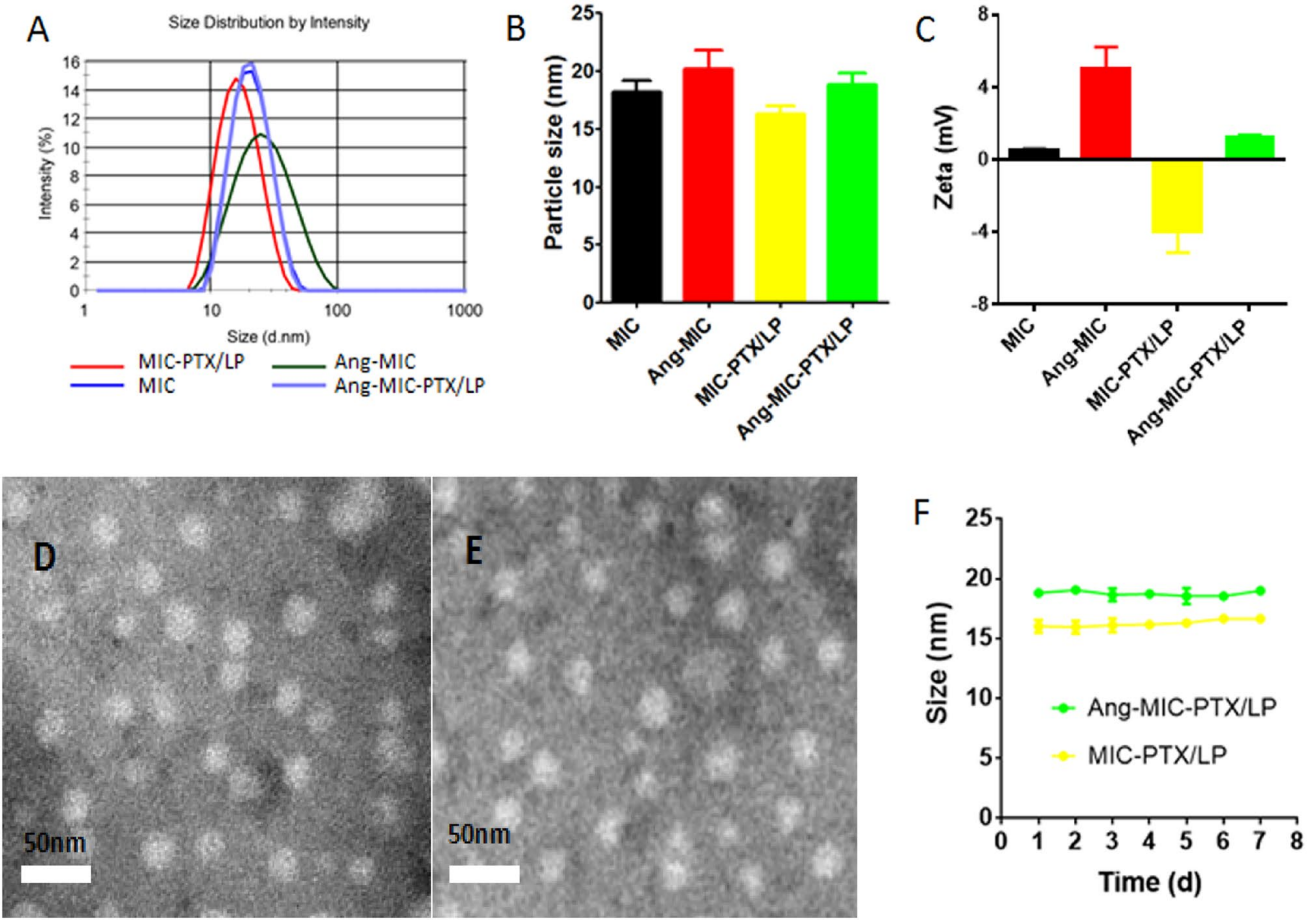
Characterization of Ang-MIC-PTX/LP. (**A**) Size distribution, (**B**) average size and (**C**) zeta potential of different micelles. TEM images of (**D**) MIC-PTX/LP and (**E**) Ang-MIC-PTX/LP. (**F**) Stability of MIC-PTX/LP and Ang-MIC-PTX/LP stored at 4 °C for 7 days (n = 3).

To achieve targeting ability, the micelles were then modified with Angiopep 2. As shown in Fig. 1A,B, the micelles could successfully co-load PTX and LP with narrow size distribution and small particle size. The conjugation of Angiopep-2 led to slight increase of the size and PDI value, probably because of the relatively large molecular weight of Angiopep-2. As previously reported, micelles with particle smaller size showed longer half-life in blood. Moreover, the smaller size was beneficial for tumor passive targeting through the enhanced permeability and retention (EPR) effect^20^. Figure 1C showed the changes of surface charge of micelles, and it also suggested the successful drug loading and conjugation of targeting molecule. The morphology and size of nanoparticles were further examined by Transmission electron microscopy (TEM). TEM images (Fig. 1D,E) revealed that both MIC-PTX/LP and Ang-MIC-PTX/LP were uniform and spherical particles with nanometer-size consistent with results measured by DLS. The micelles showed good stability without obvious size changes during the whole study period (Fig. 1F).

The amount of Angiopep-2 on micelle surface was examined by BCA assay, and the ratio of Angiopep-2 to COOH-PEG-PLA was calculated to be 1:1.07. The successful modification of targeting molecule Angiopep-2 was necessary for the active targeting capability of the DDS.

**Figure 2:**
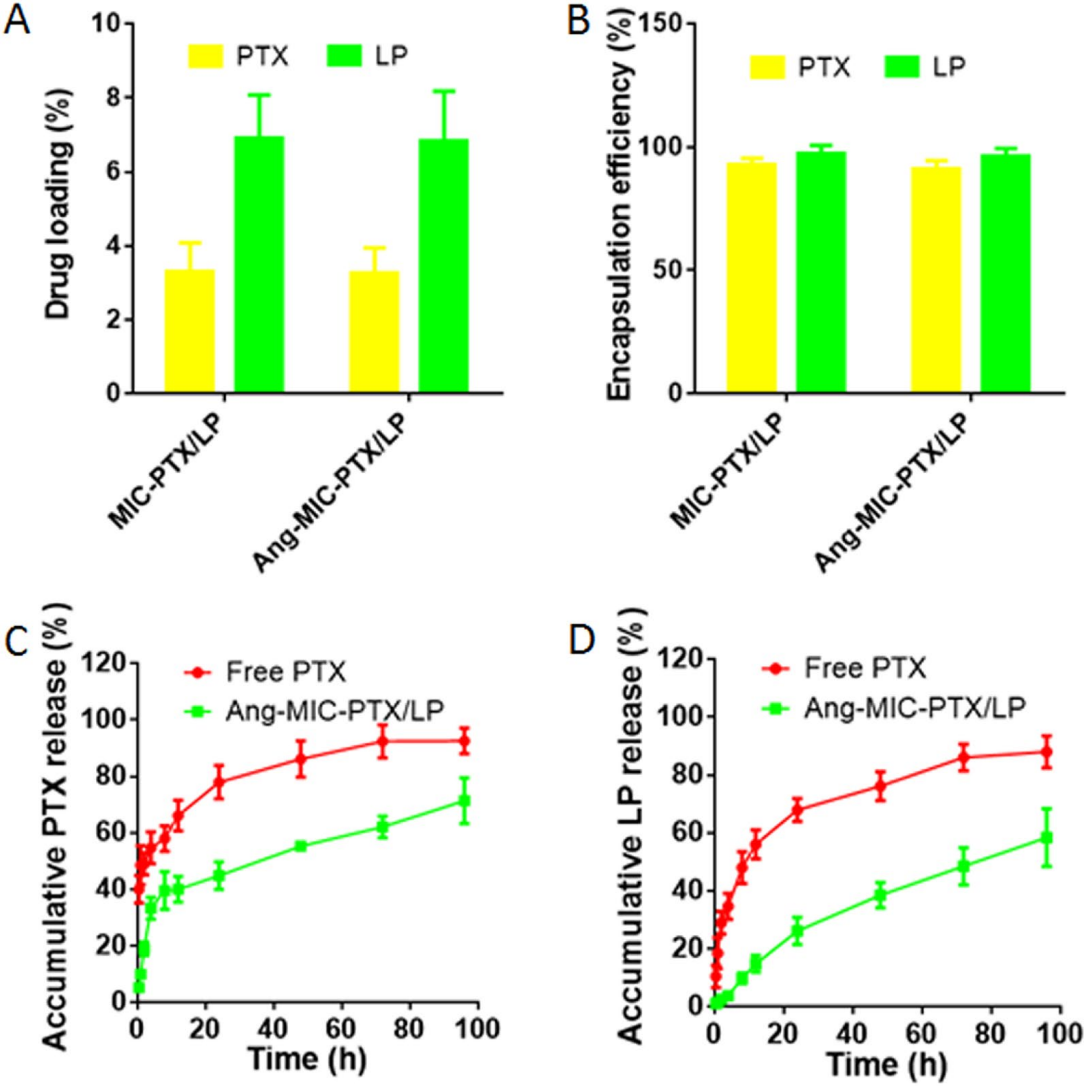
Drug loading capability and drug releasing profile of Ang-MIC-PTX/LP. (**A**) Drug loading of PTX and LP in MIC-PTX/LP and Ang-MIC-PTX/LP. (**B**) Encapsulation efficiency of PTX and LP in MIC-PTX/LP and Ang-MIC-PTX/LP. LP Released profile of (**C**) PTX and (**D**) from Ang-MIC-PTX/LP in 5% FBS containing 0.1% Tween 80 (n = 3).

The LC% and EE% of PTX and LPTN in micelles were measured by HPLC assay. The results showed that mPEG–PLA micelles had a good capacity for PTX and LPTN, with LC of 2.9 ± 0.8% and 6.82 ± 1.16%, EE of 95.97 ± 3.17% and 91.21 ± 2.83% for PTX and LPTN respectively (Fig. 2A,B). It indicated that paclitaxel and lapatinib were well co-wrapped in micelles.

In Fig. 2C,D, both free PTX and LP showed nearly complete release within 72 h. In contrast, Ang-MIC-PTX/LP showed significantly sustained release for PTX (70%) and LP (60%) even at the end of the study (96 h).

### The ability of BBB penetration

**Figure 3:**
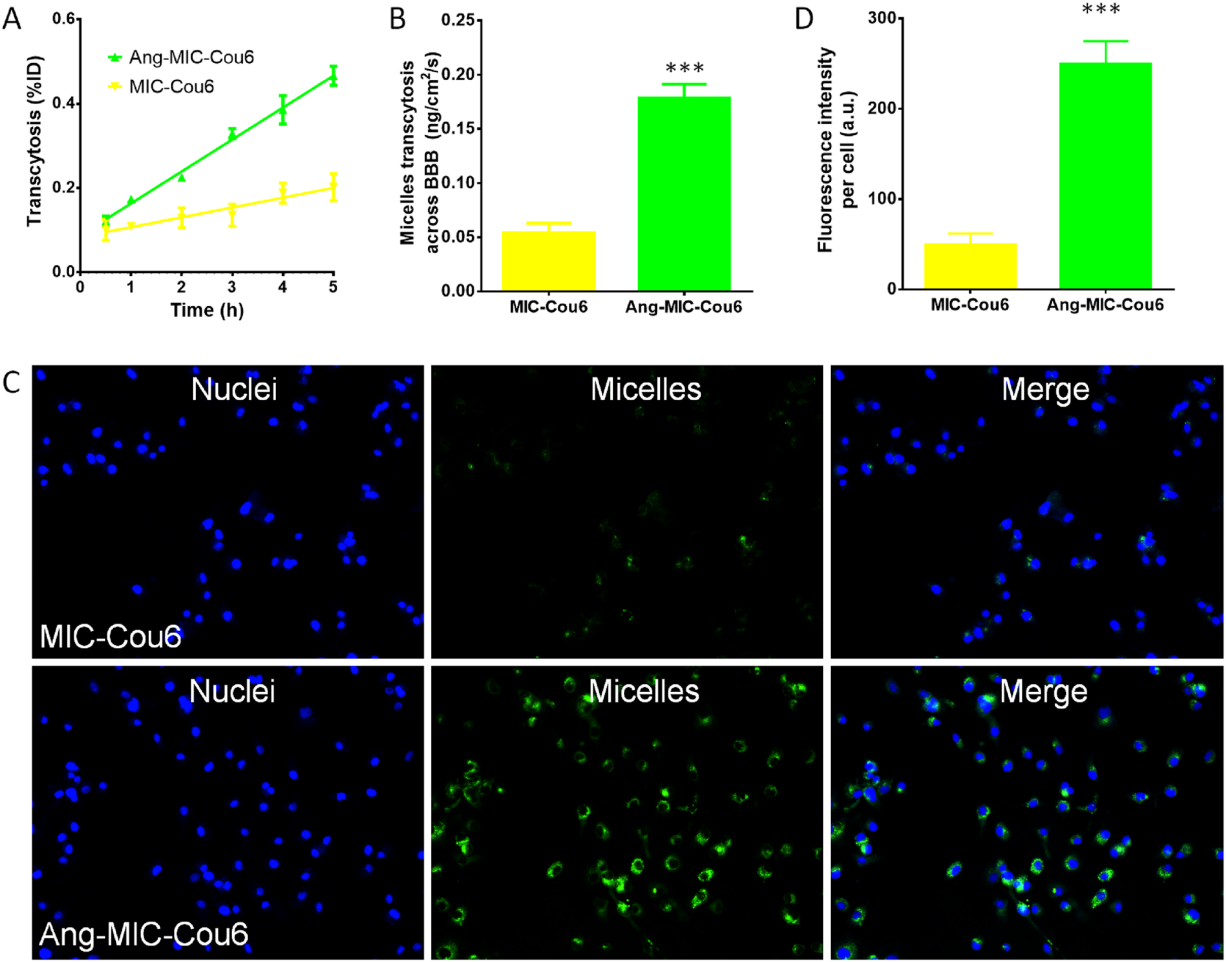
Transport of coumarin-6-labeled Ang-MIC acrossed the BBB model in vitro. (**A**) The signal of micelles transported across the BBB was plotted with time. (**B**) Transport rate of micelles acrossed the BBB. The uptake of micelle by Skbr-3 cells in the receptor cells (**C**) imaged by a fluorescence scope and (**D**) quantitatively determined by flow cytometry.

In vitro BBB model was established to evaluate the BBB penetration ability of Ang-MIC. The fluorescence dye (coumarin-6) label micelles (MIC-cou-6 and Ang-MIC-cou-6) were used in this study. The tumor cells in lower compartment was observed by a confocal microscope. Increase fluorescence signal in tumor cells was observed in Ang-MIC-cou-6 group (Fig. 3C). In addition, the cellular uptake of MIC-cou-6 and Ang-MIC-cou-6 in SKBr-3 cells on lower chamber was measured quantitatively by flow cytometry (Fig. 3D), which was consistent with confocal images. It revealed that the modification of Angiopep-2 significantly increased transcytosis of the micelles across the BBB model.

To further verify the effects of these micelles, we confirmed the drug effect in the body of tumor-burdened nude mice. Herein, we clearly observed that Ang-MIC-Dir (Fig. 3B) can more fleetly pass through the BBB, thus reaching the brain tissue at an earlier date than MIC-Dir (Fig. 3A). It can also persist in the targeted site for a long time which directly proves the excellent brain targeting function of Ang-MIC-Dir.

**Figure 4:**
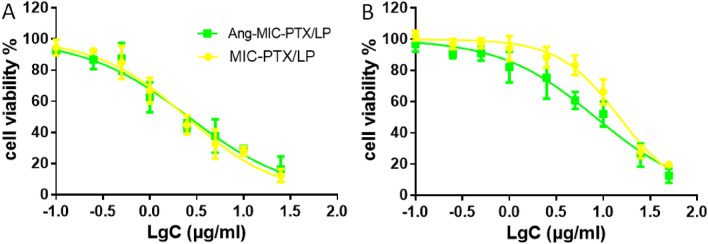
Cell viability of (**A**) SKBr-3 cells and (**B**) 4T1 cells after treated with different concentrations of Ang-MIC-PTX/LP. MIC-PTX/LP group was used as control.

The cytotoxicity of MIC-PTX/LP and ANG-MIC-PTX/LP on both cells was investigated by MTT assay (Fig. 4A). For Skbr3 cells, the IC50 for MIC-PTX/LP is 2.455 (95% CI 1.830–3.294) and the IC50 for ANG-MIC-PTX/LP is 2.093 (95% CI 1.511–2.899) (Fig. 4A). For 4T1 cells, the IC50 for MIC-PTX/LP is 15.110 (95% CI 10.880–20.99) and the IC50 for ANG-MIC-PTX/LP is 9.238 (95% CI 6.402–13.33) (Fig. 4B). For HER2-positive cells, the complex micelles were more cytotoxic, highlighting the targeting of lapatinib.

### Brain tumor targeting ability

**Figure 5:**
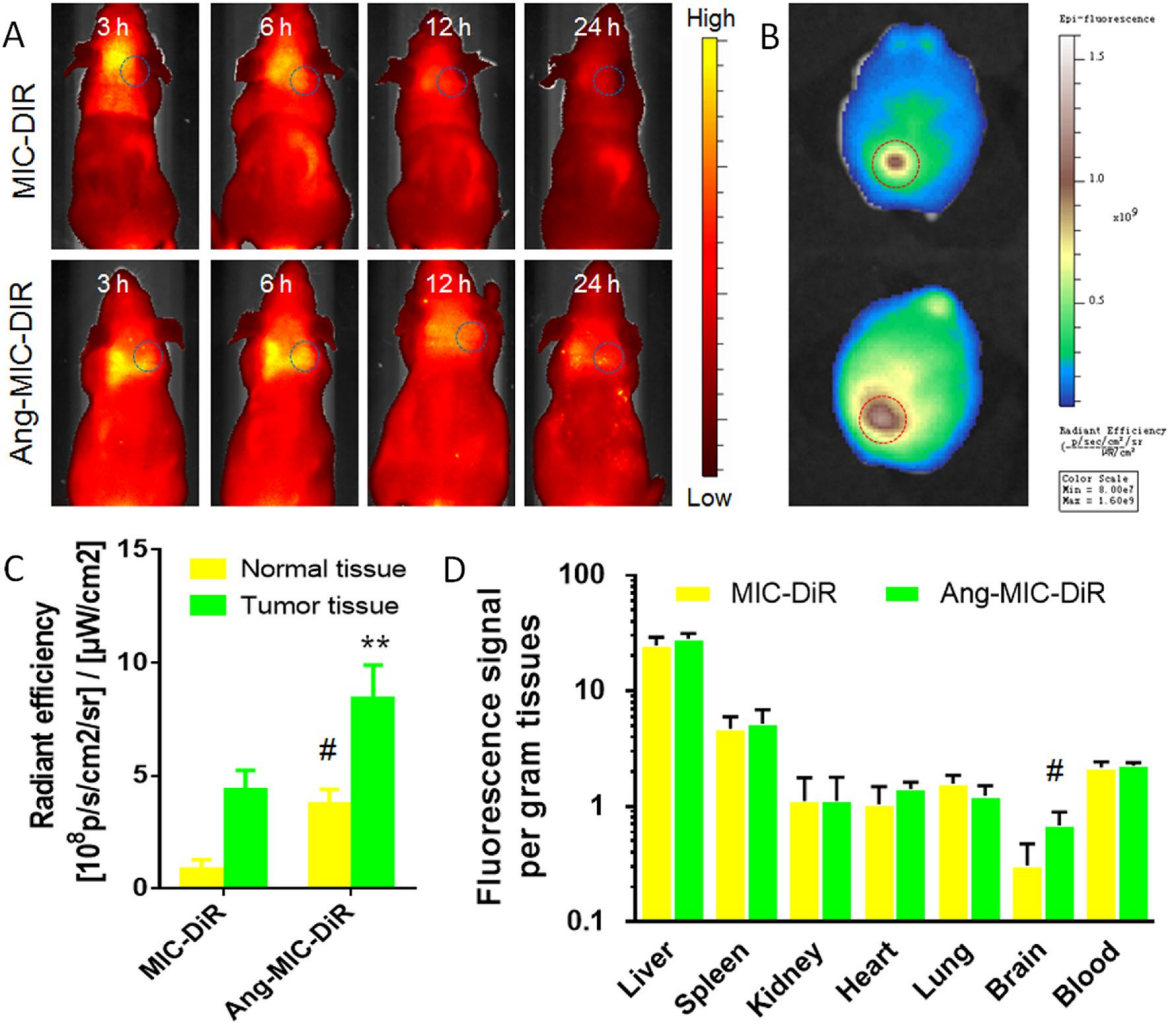
Brain tumor accumulation of Ang-MIC. (**A**) In vivo imaging of tumor-bearing mice administrated with DiR-labeled Ang-MIC. (**B**) Ex vivo imaging of brains and (**C**) semi-quantification results of fluorescence of brain tumor tissues 24 h post micelle injection. (**D**) Biodistribution of Ang-MIC 24 h post micelle injection (n = 3). ^#^*P* < 0.05, **P < 0.01, compared with MIC.

To confirm whether ANG-MIC could penetrate the BBB and target tumor in vivo, breast tumor-bearing nude mice model was used. The model mice were intravenously injected with DiR-MIC or DiR-Ang-MIC. The DiR-Ang-MIC showed significantly higher accumulation efficiency both in the brain compared with DiR-MIC group (Fig. 5A). Especially, the results of ex vivo imaging of brains confirmed that the highest fluorescence signal existed in the tumor region (Fig. 5B,C). Biodistribution results were shown in Fig. 5D, and micelles were mostly distributed in liver and spleen like other types of nanoparticles.

### In vivo anti-tumor ability

**Figure 6:**
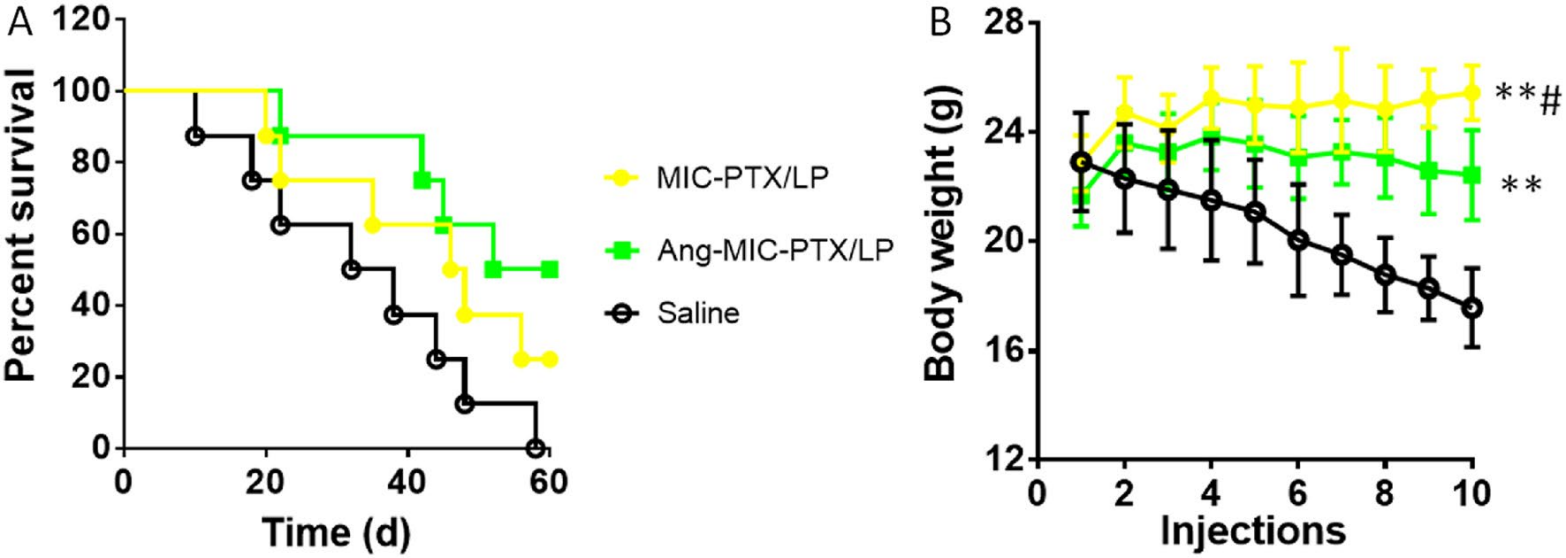
Anti-tumor efficacy of Ang-MIC-PTX/LP. (**A**) Survival curves (n = 8) and (**B**) body weight of tumor-bearing mice treated with ten cycles of different formulations at 3, 6, 9, 12, 15, 18, 21, 24, 27 and 30 days after Skbr-3 cells inoculation. The dose of PTX and LPT was 2.5 mg/kg and 5 mg/kg, respectively.**^#^*P* < 0.05, **P < 0.01, compared with saline.

Compared with MIC-PTX/LP group and saline group, Ang-MIC-PTX/LP could significantly improve survival rate. A combined effect of active targeting and passive targeting could be the main reason for the benefits (Fig. 6). However, comparing two of them, we found that the difference between the ANG group and the MIC group was not statistically significant, nor was the difference between the MIC group and the control group.

The effects of brain metastatic tumor resistance of these two micelles were investigated by monitoring the survival time of a tumor-burdened nude mice. The median survival time was exhibited in Table 1.

**Table 1:** The median survival time of tumor-bearing mice treated with different preparation.

Group	Median (day)	Log-rank test	ISTC
Saline	35		–
MIC-PTX/LP	47	^a^	34.3%
Ang-MIC-PTX/LP	56	^a^^,^^b^	60.0%

^a^*P* < 0.05, ^b^*P* < 0.05, compared with saline and MIC-PTX/LP, respectively.

The increases in survival times (%) were compared to control (ISTC).

## Discussion and conclusion

HER-2 over-expressing breast cancer signifies a poor prognosis and about half of the HER-2 over-expressing patients with advanced breast cancer would develop brain metastases^21^. Due to the barrier of the blood–brain barrier, traditional drug treatments are ineffective for breast cancer brain metastases, and surgery or radiotherapy is the priority at this time^22^. However, most advanced patients have poor physical conditions, multiple underlying diseases and numerous brain metastases, which make it difficult to tolerate surgery, or meet radiotherapy standards^23^. Traditional drugs are difficult to pass through the blood–brain barrier due to molecular structure and other reasons, and even if they enter the brain tissue, without efficiently targeting the tumor tissue, the indiscriminate attack on the surrounding normal tissues will also produce serious side effects, such as brain tissue edema and vomiting. Even coma^24^. Most importantly, low-efficiency treatment can easily lead to drug resistance in tumor cells, thereby shortening the survival time of cancer patients.Therefore, the choice of effective drugs and the brain targeted delivery system has become the preferred solution to overcome this problem.

PTX is one of the most effective chemotherapy drugs for breast cancer. However, due to primary or secondary resistance reaction, PTX does not work in patients with advanced breast cancer. Up until now, it has been reported that LPNT could make HER-2 overexpressing breast cancer more sensitive to PTX^6–11^. Hence, we constructed a novel micellar delivery system combing LPTN and PTX so as to achieve an enhanced antineoplastic effect.

At present, there have been many mature drug delivery systems for brain targeting, such as nano-drug delivery systems, but most of them are macromolecular polymers, and mainly used to treat gliomas^25,26^. Polymer glue Bundles have been developed to treat cerebral ischemia–reperfusion injury^27^ and liposome delivery systems have also been widely used in the treatment of gliomas^28–30^. All of the above are based on the targeting properties of Angiopep-2, which is widely used and recommended by the Food and Drug Administration (FDA) to pass through the BBB. ANG-1005, which has been approved by the FDA for marketing, is Angiopep-2 connected to paclitaxel as a brain-targeted drug delivery system^31–34^. However, the connection between Angiopep-2 and the drug mainly relies on peptide bonds, which are not stable. The current solution is to modify the drug with PEG to reduce the first pass effect of the drug in the body. Studies have shown that nanoparticles smaller than 35 nm can penetrate the blood–brain barrier more easily. Our study used a drug delivery system with a particle size of 20 nm, which has been shown to have superior ability to penetrate the blood–brain barrier in previous studies^35,36^. We have developed a dual-targeted nanomedicine to obtain superior cell selectivity, cell uptake and tumor penetration ability, which shows great advantages in tumor targeted therapy. From the above researches, when designing dual-targeted nanomedicine, the following aspects need to be considered. First of all, the size and stability of the particles largely determine the delivery efficiency of the drug, which is also a prerequisite for the drug to perform its function at the tumor site. Secondly, when applied in vivo, the actual targeting effect of this nanomedicine becomes very important. Due to the complex tumor microenvironment, its in vitro targeting effect may not achieve the best results in vivo. Therefore, it is necessary to further effectively monitor the tumor microenvironment in order to optimize the targeting effect of dual-targeting micelles. Based on clinical needs and the current development of materials science, we developed a dual-targeted micelle loaded with paclitaxel and lapatinib for the treatment of brain metastases HER2-positive breast cancer. Our innovation lay in the loading of two drugs at the same time. The special ratio of these two drugs was very important for successfully killing breast cancer brain metastases. Since there is no compound preparation of the two drugs at present, we have no direct reference. Starting from the clinical dosage, we conducted a lot of experiments to achieve the best ratio and the ratio of PTX and LPTN was determined to be 1:2, in order to make the encapsulation rate of the drug and the amount of drug loading meet the experimental requirements. In vitro experiment of the two drugs mixed in proportion, the IC50 value is lower than the amount of the two drugs used alone. Another major innovation of this experiment was that the micelles made of poly(ethylene glycol)-poly(lactide) (PEG-PLA) were further modified with Angiopep-2 (Ang-MIC), which could simultaneously target brain and tumor. After the drug crossed the blood–brain barrier, it would accurately hit the tumor tissue and reduce the damage to the normal brain tissue, which would be greatly beneficial to prolong the survival. In our research, the brain delivery and brain metastasis targeting capabilities of this carrier system had been confirmed in vitro and in vivo, making the delivery of chemotherapeutic drugs more controllable and effectively reducing the toxicity of these drugs to normal tissues.

For HER2-positive breast cancer, anti-HER2 drugs, such as trastuzumab and lapatinib, are routinely used in clinical practice, but the effect of the treatment of brain metastases is poor due to the blood–brain barrier. In addition to the aforementioned ANG-1005, there were also large-molecule monoclonal antibodies, small-molecule TKIs (tyrosine kinase inhibitors) and ADCs (antibody–drug conjugates) can be applied to HER-2 positive breast cancer brain metastases. However, they were not purely brain-targeted, and more importantly would cause a certain degree of damage to the body^22,37–39^. The dual-targeted drug delivery system prepared by us had significantly stronger anti-brain metastasis efficacy than single-agent paclitaxel or lapatinib. The survival period of nude mice was also significantly prolonged. Our study proved the safety and effectiveness of this delivery system and effectively solved clinical problems.

However, the potential of compound micelles was still quite unstable and difficult to measure. We also found that the MIC-PTX/LP group exhibited symptoms such as ascites and hepatomegaly during the period of medication. We speculated that the micelles may have caused damage to the endothelial system. The literature has reported that because the plasma membrane surface of compound micelles was negatively charged, positively charged micelles can enter the BBB through electrostatic action^40^. But the positive charge should not be too much, otherwise it would cause damage to the body. Fortunately, in vivo experiments, there was no significant decrease in body weight of nude mice after the drug was administered, so the drug was considered to be safety.

In this sdudy, we only developed a murine-derived nude mouse model of triple-negative breast cancer cells, and lacked of a human-derived triple-negative breast cancer model for more accurate comparisons. We would further study the treatment of human breast cancer models with similar growth rates to obtain more accurate research results in future.

In conclusion, this experiment proves that nude mice with brain metastases tumor can survive longer after receiving the treatment by the composite micelles. Thus, it shows a bright future and promising result for the study and application of this kind of nano-drug for oncotherapy. However, the underlying mechanisms of the synergistic effect of this dual targeted micelles need be carefully investigated and clarified. Besides, more efforts should be exerted to the design, production and control facets of dual targeted micelles for combinational therapy to facilitate possible clinical translation.
